# Prediction model of in-hospital mortality risk in intensive care unit patients with cardiac arrest: a multicenter retrospective cohort study based on an ensemble model

**DOI:** 10.3389/fcvm.2025.1582636

**Published:** 2025-05-20

**Authors:** Li Liu, Wei-Wei Lai, Bo-Wen Li, Shu-Hang Wang, Mu-Ming Yu, Yan-Cun Liu, Yan-Fen Chai

**Affiliations:** ^1^ Department of Emergency Medicine, Tianjin Medical University General Hospital, Tianjin, China; ^2^ College of Environmental Science and Engineering, Nankai University, Tianjin, China

**Keywords:** in-hospital cardiac arrest, ensemble learning, clinical prediction model, machine learning, web application

## Abstract

**Background:**

In-hospital cardiac arrest (IHCA) is a major adverse event with a high death risk. Machine learning (ML) models of prognosis in cardiac arrest (CA) patients have been established, but there are some interferences in their clinical application. This study developed an ensemble learning (EL) model based on clinical information to predict IHCA patient death risk.

**Methods and results:**

This retrospective cohort study used data from the Medical Information Mart for Intensive Care IV (MIMIC-IV) database and eICU Collaborative Research Database. Patients (age ≥ 18 years) with CA based on the ICD-9/10 code were included. Eight candidate ML models were selected for soft voting ensemble. Features were sequentially eliminated based on feature importance scoring to reduce input complexity without compromising model performance. The final model was externally validated with the MIMIC-IV database and deployed as a web application. Overall, 4,068 patients were included. In the internal validation cohort, the EL model exceeded single ML models with an accuracy of 0.842, precision of 0.830, recall of 0.839, F1 score of 0.835, and AUC of 0.898 and showed better calibration across the spectrum of survival probabilities. Furthermore, there is no obvious decline in the prediction performance of the EL model with the top seven features (HCO_3_^−^, Glasgow Coma Scale, white blood cell count, international normalized ratio, hematocrit, body temperature, and blood urea nitrogen) retained. In external validation, the performance slightly decreased but remained acceptable for deploying a clinically feasible web application.

**Conclusion:**

The EL model outperformed single ML models in predicting IHCA patient death risk. The identified seven key features enabled the parsimonious EL model to reliably estimate the death risk.

## 1 Introduction

In-hospital cardiac arrest (IHCA) is a major adverse event, affecting >200,000 people annually in the USA, and is associated with a poor survival rate of approximately 20% (1, 2). Efforts to improve the survival of IHCA patients have focused on the timeliness and quality of resuscitation, e.g., early defibrillation or cardiopulmonary resuscitation (CPR) has been proven to improve the success of return of spontaneous circulation (ROSC) (3, 4). Given the high incidence of and low survival of IHCA, early and accurate identification of IHCA patients with high death risk will help clinicians make optimal clinical decisions and improve the patient prognosis.

Biomarkers can be used to monitor the progress and predict the prognosis of IHCA patients, such as neuron-specific enolase (NSE), S100B protein (S100B), TNF-α, IL-6, high-sensitivity C-reactive protein (hsCRP), and endothelin-1 (5–9). However, the long testing time and complexity of the testing process make them difficult to promote in clinical settings. The scoring systems have been routinely used to evaluate the overall condition and predict patient prognosis in clinical settings, such as sequential organ failure assessment (SOFA), the National Early Warning Score 2 (NEWS2), and the cardiac arrest risk triage (CART) (10, 11). In addition, statistical methods have been widely used for survival prediction in CA patients. Shang et al. (12) applied logistic regression (LR) to establish a prediction model for sudden cardiac death (SCD) in 262 hospitalized patients, which demonstrated good prediction performance with an AUC of 0.774. However, scoring systems and statistical models are limited by the amount of clinically available features that are ignored and the assumption of linear relationships between clinical features, which fundamentally makes them not ideal in terms of accuracy and reliability (13, 14).

Machine learning (ML), as a typical form of artificial intelligence, offers methods for deciphering complex non-linear relationships, achieving trend predictability, and discovering new knowledge hidden behind big data (15, 16). ML models have demonstrated effectiveness in capturing potential associations between clinical features and IHCA death risk (17). Sun et al. (11) established a LASSO model to predict IHCA death risk. Deep learning (DL), as a subset of ML, also demonstrated advantages in capturing temporal information representation of longitudinal variables and extracting features from electrocardiography (ECG) data for ICU death and sudden cardiac death (SCD) risk prediction (18, 19). However, recurrent neural networks (RNNs) struggle to handle irregular time-series data caused by emergency resuscitation, while convolutional neural networks (CNNs) generally only consider ECG is not entirely sufficient (20, 21). The mismatch with clinical data structure and clinical urgency limits the clinical practicality of DL methods.

Moreover, pure ML methods need to further improve the prediction capability while reducing input complexity, which dictates the clinical utility of prediction models since any clinician will be unable to enter data on too many variables during ongoing CPR (22). Studies show that a single ML model can be outperformed by a “committee” of individual models, which is called ensemble learning (EL) (23). EL is a powerful ML paradigm that assembles multiple models to enhance model prediction performance and generalization capability (24). Ensemble models have been proven to be effective as they can significantly reduce the bias of individual models and improve diagnostic accuracy by combining the predictions of multiple basic models (25–27). To optimally support clinicians, interpretability of the results is also essential besides prediction accuracy. The Shapley Additive exPlanation (SHAP) method can provide both local and global interpretation and excellent visualization capabilities, which can be applied to break through the “black box” in any ML model and help clinicians evaluate the rationality of the model prediction (28, 29).

This study used the EL strategy to develop a death risk prediction model for IHCA patients. A total of 54 candidate clinical features in 1,472 and 2,596 cases of IHCA were extracted from two databases for internal and external validation, respectively. The key factors affecting the IHCA death risk were explored via the Gini impurity and SHAP method, and then the optimal feature subset was identified to reduce input complexity without compromising model performance to help clinicians quickly and reliably estimate the death risk of IHCA patients. The final model was deployed into a web application for practical application in clinical settings.

## 2 Materials and methods

### 2.1 Data collection

This was a retrospective cohort. The data used in this study were extracted from the Medical Information Mart for Intensive Care IV (MIMIC-IV) database (v2.2) and the eICU Collaborative Research Database (eICU-CRD). The eICU-CRD is a multicenter database of 335 units at 208 hospitals located throughout the USA between 2014 and 2015. The authors who acquired data from the databases completed the course and obtained certification (No. 61529195). The MIMIC-IV consists of data from the Beth Israel Deaconess Medical Center from 2008 to 2019. The eICU-CRD and MIMIC-IV database received ethical approval from the Institutional Review Boards and the Massachusetts Institute of Technology. As the two databases did not contain identified health information, a waiver of informed consent was included in the approval. The eICU-CRD was used for model development and internal validation to check the repeatability, while the MIMIC-IV database was used as the external validation cohort to assess model transportability and generalizability.

### 2.2 Patients

**Figure 1:**
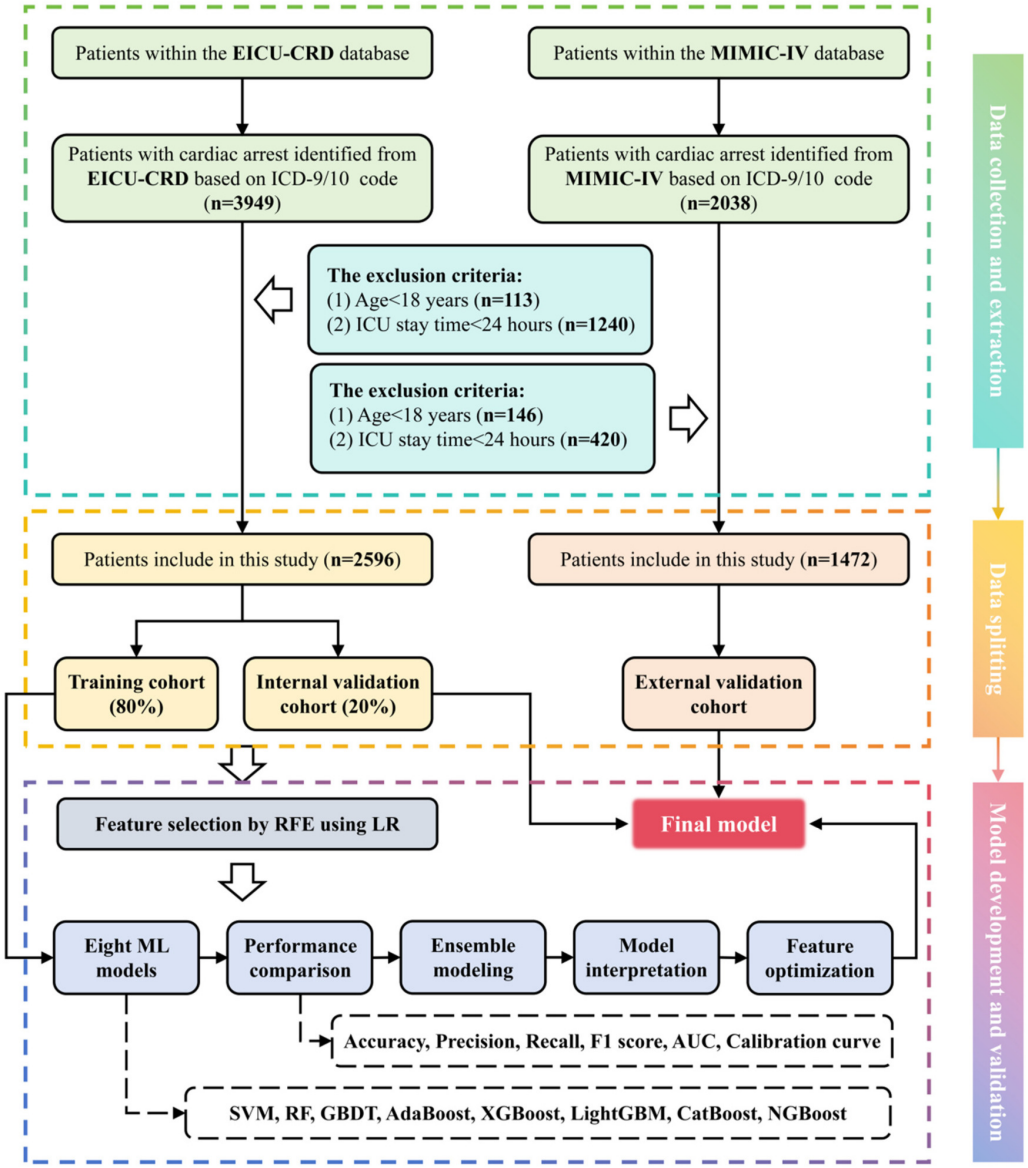
Flowchart for developing a death risk prediction model for IHCA patients.

All Patients in the MIMIC-IV database and eICU-CRD who met the following criteria were included in this study: (1) patients who were 18 years old or older and (2) patients with cardiac arrest identified based on the ICD-9/10 code. The exclusion criteria were as follows: (1) patients under the age of 18 and (2) those with ICU stay time of no >24 h. For patients with multiple admissions or a history of ICU stays, only those with their first ICU experience during their first admission are included. The flowchart showed the selection of patients in this study (Figure 1). Finally, 4,068 people were included in the study.

### 2.3 Data extraction

The clinical data of patients with MIMIC-IV and eICU-CRD IHCA were extracted using PostgreSQL tools in pgAdmin and Navicat Premium. The prediction model only included the clinical and laboratory variables on the first day of ICU admission. If the patient received more than one vital sign measurement or laboratory test on the first day of admission, the average values were used for subsequent analysis. Patient characteristics were collected, including emergency department (ED) admission, age, sex, height, and weight. Information on comorbidities, such as myocardial infarct, congestive heart failure (CHF), peripheral vascular disease, cerebrovascular disease, dematia, chronic pulmonary disease, immune system disease, peptic ulcer disease, diabates mellitus (DM), paraplegia, and renal disease, were extracted based on the International Classification of Diseases coding system. Vital signs were collected, including heart rate (HR), systolic blood pressure (SBP), diastolic blood pressure (DBP), mean blood pressure (MBP), respiratory rate (RR), SPO_2_, and body temperature. Laboratory tests included hemoglobin (HB), hematocrit (HCT), platelet, red blood cell count (RBC), white blood cell count (WBC), prothrombin time international normalized ratio (INR), prothrombin time (PT), partial thromboplastin time (PTT), creatinine, blood urea nitrogen (BUN), aspartate aminotransferase (AST), glutamic pyruvic transaminase (ALT), alkaline phosphatase (ALP), ceatine kinase (CK), creatine kinase isoenzymes (CKMB), brain natriuretic peptide (BNP), glucose, K^+^, Na^+^, Ca^2+^, Cl^−^, anion gap, pH, HCO_3_^−^, and lactate. The extracted treatment information included ventilation, epinephrine, dopamine, and vasopressin. The extracted marking systems included the Glasgow Coma Scale (GCS) and Charlson Comorbidity Index (CCI). The primary outcome of the study was in-hospital death, defined as the vital status of the patient at discharge.

### 2.4 Data processing and analysis

The missing values in the raw dataset from eICU-CRD were handled using the *K*-nearest neighbor (KNN) imputer. KNN imputation leverages local patient similarity to preserve subgroup-specific distributions, which avoids the linear assumptions of regression methods and maintains feature correlations better than mean imputation. The imputed dataset was randomly divided into the training cohort (80% of the data) and the internal validation cohort (20% of the data). The synthetic minority oversampling technique (SMOTE) was conducted to generate synthetic samples to reduce class imbalance in the raw training cohort to enhance the robustness in prediction and extrapolation (30). Furthermore, all data values of continuous features were normalized to the range of 0–1 according to Equation 1 to eliminate dimensional differences and hasten the learning process (31). The categorical features were converted into numerical variables by label encoding to unify the data format (32).

(1) $$x=\frac{x_{\mathrm{raw}}-x_{\min}}{x_{\max}-x_{\min}}$$

where *x* is the normalized value, $x_{\mathrm{raw}}$ is the raw value that has not been normalized, and $x_{\min}$ and $x_{\max}$ are the minimum and the maximum values in the raw dataset, respectively.

### 2.5 Ensemble modeling methodology

Eight ML models, including support vector machine (SVM), random forest (RF), gradient boosting decision tree (GBDT), adaptive boosting (AdaBoost), extreme gradient boosting (XGBoost), light gradient boosting machine (LightGBM), categorical boosting (CatBoost), and natural gradient boosting (NGBoost), were initially selected as candidate basic models for ensemble modeling. Although CNNs/RNNs excel in many ML applications, clinical prediction based on tabular data classification is still dominated by the bootstrap aggregating (Bagging) algorithm and boosting algorithm, largely due to their short training time and robustness (33, 34). Importantly, IHCA is an acute, time-sensitive event where clinicians need immediate predictions based on baseline data, not long-term trends, which drives the exclusion of more advanced models such as CNNs or RNNs.

During model training, fivefold cross-validation and grid search were performed in the training cohort to achieve the optimal hyperparameters for each model. Note that cross-validation should be performed after applying SMOTE. The accuracy (Equation 2), precision (Equation 3), recall (Equation 4), F1 score (Equation 5), and the area under the receiver operating characteristic (ROC) curve (AUC) were used to evaluate the prediction performance of trained models. The basic models with the best prediction performance would be used to establish an EL model for stronger robustness and diagnostic accuracy through the soft voting strategy, which weights predictions of multiple ML models based on their confidence or probability estimates to obtain a more accurate and robust final prediction.

(2) $$\mathrm{Accuracy}=\frac{\mathrm{TP}+\mathrm{TN}}{\mathrm{TP}+\mathrm{TN}+\mathrm{FP}+\mathrm{FN}}$$

(3) $$\mathrm{Precision}=\frac{\mathrm{TP}}{\mathrm{TP}+\mathrm{FP}}$$

(4) $$\mathrm{Recall}=\frac{\mathrm{TP}}{\mathrm{TP}+\mathrm{FN}}$$

(5) $$F1\;\mathrm{score}=\frac{2\times \mathrm{Precision}\times \mathrm{Recall}}{\mathrm{Precision}+\mathrm{Recall}}$$

where TP is the number of true positives, TN is the number of true negatives, FP is the number of false positives, and TN is the number of false negatives. Different metrics provide different perspectives on the performance evaluations of trained models. Accuracy provides overall correctness of the model predictions, but it may not be sufficient in imbalanced datasets. Precision is important when false positives are costly, recall is vital when missing positive cases is unacceptable, and the F1 score combines the strengths of both. AUC is calculated by integrating the ROC curve, which ranges from 0 to 1. A model with an AUC of 1 is perfect, while a model with an AUC of 0.5 is no better than random guessing.

### 2.6 Model interpretation

The SHAP method was adopted to interpret the EL model assembled by multiple diverse basic models. SHAP connects optimal credit allocation with local explanations using the classical Shapley values from game theory and their related extensions, which represent a thorough theoretical demonstration of consistent and unbiased interpretation methods (29, 35). The Shapley value $\phi_{i}$ of the feature *i* was calculated according to Equation 6 (36). The Shapley value of each feature quantified its contribution, whether negative or positive. A feature with a higher mean absolute Shapley value implied a greater impact on the model output.

(6) $$\phi_{i}=\underset{S\subseteq N/\{i\}}{\sum }\frac{|S|!(n-|S|-1)!}{n!}(\;f(S\cap \{i\})-f(S))$$

where *S* is the subsets of all features with feature *i*,f(S∩{i}) denotes the prediction by the established EL model considering feature *i*, and f(S) is the prediction without considering feature *i*. The differences among all possible subsets of S⊆n are calculated due to the dependency of the effect of withholding a feature on other features in the EL model. In addition, for decision-tree–based models, the feature importance can be assessed by the Gini impurity used for the calculation of splits in the tree. The Gini impurity is computed during training based on how much each feature decreases the weighted impurity in a tree (37, 38). This method was only adopted in the EL model consisting of decision-tree–based models, such as RF, GBDT, AdaBoost, XGBoost, LightGBM, CatBoost, and NGBoost.

### 2.7 Feature optimization

The SHAP method and Gini impurity clarified which clinical features are the most important for the death risk of IHCA patients. Each feature was ranked in descending order based on feature importance and then eliminated to determine how many features were required to reduce input complexity without compromising model performance. Furthermore, the top features were used to establish parsimonious EL models with varying numbers of features in order of feature importance. The parsimonious EL model would be retrained in each subset of eliminated features and evaluated using accuracy, precision, recall, F1 score, and AUC to determine how many features were required to achieve an acceptable model performance to deploy a clinically feasible web application.

### 2.8 Statistical analysis

Data analysis was performed using SPSS version 27.0.1. For continuous variables, the normality was assessed by the Kolmogorov–Smirnov test. The normally distributed data were presented as mean ± standard deviation, and the independent samples *t*-test was used for their between-group comparisons. The non-normally distributed data were expressed as median with interquartile range (IQR: P25, P75), and the Mann–Whitney *U* test was used for their between-group comparisons. Categorical variables were described using frequencies and percentages (%), and between-group differences were evaluated using the chi-square (*χ*²) test or corrected chi-square test. Statistical significance was set at *P* < 0.05.

### 2.9 Webpage deployment tool based on Streamlit framework

To facilitate the utility of the model in clinical settings, the final prediction model was implemented into a web application based on the Streamlit Python-based framework. When the values of corresponding features from the final model are provided, the application can return the IHCA patient death risk and its probability.

## 3 Results

### 3.1 Baseline characteristics

A total of 5,987 patients were diagnosed with CA on admission according to ICD-9/10, and a total of 1,919 patients were excluded according to the exclusion criteria. Finally, 4,068 patients were included in our study, of which 1,472 patients were from the MIMIC-IV database and 2,596 patients were from the eICU-CRD, as shown in Figure 1. Statistical differences of clinical features between the survival and death groups using the eICU-CRD as the internal validation cohort are shown in Table 1, while differences using the MIMIC-IV database as the external validation cohort are shown in Supplementary Table S1. In the raw dataset, the proportion of female and ED admissions was lower in the death group (*P* < 0.005). Body temperature, platelet count, CK, PH, HCO_3_^−^, and GCS values were lower in the death group (*P* < 0.005). Patients who died also were older, had higher incidence of peripheral vascular disease, dementia, and chronic pulmonary disease, and had elevated levels of HR, RR, WBC, INR, PT, creatinine, BUN, ALT, AST, ALP, CKMB, BNP, K^+^, Na^+^, Ca^2+^, Cl^−^, anion gap, glucose, and lactate. In addition, there was a significantly higher proportion of ventilatory treatment, epinephrine use, dopamine use, and vasopressin use (*P* < 0.005). Moreover, Supplementary Table S2 indicates that 36 out of 54 initial features showed significant differences (*P* < 0.005) between the MIMIC-IV database and eICU-CRD, implying that MIMIC-IV can be effectively used for external validation to test the model’s transportability and generalizability.

**Table 1:** Baseline characteristics of the patients.

Feature	Raw dataset (*n* = 2,596)		
	Survival (*n* = 1,440)	Death (*n* = 1,156)	*P*-value
Patient characteristics			
ED admission, *n* (%)	600 (41.70)	541 (46.80)	0.009
Age (years old) [median (IQR)]	65.00 (54.00, 74.00)	66.00 (76.00, 55.00)	0.007
Mean, *n* (%)	875 (60.76)	658 (56.92)	0.048
Height (cm) [median (IQR)]	170.20 (162.60, 177.80)	170.20 (162.60, 177.80)	0.239
Weight (kg) [median (IQR)]	81.80 (70.00, 99.80)	83.60 (68.10, 119.48)	0.795
Comorbidities, *n* (%)			
Myocardial infarct, *n* (%)	179 (12.43)	137 (11.85)	0.654
CHF, *n* (%)	266 (18.47)	246 (21.28)	0.074
Peripheral vascular disease, *n* (%)	67 (4.65)	86 (7.44)	0.003
Cerebrovascular disease, *n* (%)	27 (1.88)	22 (1.90)	0.958
Dementia, *n* (%)	32 (2.22)	48 (4.15)	0.005
Immune system disease, *n* (%)	30 (2.08)	24 (2.08)	0.990
Chronic pulmonary disease, *n* (%)	192 (13.33)	227 (19.64)	<0.001
Peptic ulcer disease, *n* (%)	21 (1.46)	17 (1.47)	0.979
DM, *n* (%)	495 (34.38)	412 (35.64)	0.502
Paraplegia, *n* (%)	109 (7.57)	109 (9.43)	0.090
Renal disease, *n* (%)	241 (16.74)	212 (18.34)	0.285
Vital signs			
HR (beats/minute) [median (IQR)]	81.09 (69.73, 92.71)	82.23 (69.82, 97.37)	<0.001
SBP (mmHg) [median (IQR)]	114.19 (105.88, 126.29)	115.09 (103.81, 128.60)	0.441
DBP (mmHg) [median (IQR)]	65.17 (58.94, 73.32)	64.75 (58.00, 72.64)	0.191
MBP (mmHg) [median (IQR)]	79.28 (72.79, 88.55)	79.06 (71.57, 88.30)	0.316
RR (beats/minute) [median (IQR)]	18.50 (16.37, 21.61)	21.00 (17.96, 24.80)	<0.001
SPO_2_ (%) [median (IQR)]	98.09 (96.37, 99.32)	98.09 (95.89, 99.35)	0.076
Body temperature (°C) [median (IQR)]	36.70 (36.04, 37.06)	36.00 (33.52, 36.86)	<0.001
Laboratory tests			
HB (g/dl) [median (IQR)]	8.97 (8.73, 10.48)	9.77 (9.11, 12.96)	0.796
HCT (%) [median (IQR)]	29.49 (26.40, 31.36)	31.55 (27.71, 43.20)	0.367
RBC (10^12^/L) [median (IQR)]	3.55 (3.14, 4.34)	4.07 (3.42, 5.55)	0.775
WBC (10^9^/L) [mean (SD)]	13.54 (1.13)	16.73 (1.33)	<0.001
Platelet (10^9^/L) [median (IQR)]	258.83 (224.60, 286.09)	205.71 (176.06, 263.10)	<0.001
INR [median (IQR)]	1.25 (1.10, 1.39)	1.31 (1.12, 2.75)	<0.001
PT (s) [median (IQR)]	12.95 (11.86, 14.75)	13.60 (11.31, 35.83)	<0.001
PTT (s) [median (IQR)]	33.00 (25.95, 45.81)	35.26 (26.13, 53.67)	0.557
Creatinine (mg/dl) [median (IQR)]	1.30 (0.72, 4.38)	1.63 (1.22, 2.58)	<0.001
BUN (mg/dl) [mean (SD)]	31.65 (7.21)	34.44 (3.84)	<0.001
AST (U/L) [median (IQR)]	82.00 (67.25, 253.63)	87.00 (65.50, 209.00)	<0.001
ALT (U/L) [median (IQR)]	60.25 (27.00, 152.00)	73.75 (31.25, 124.63)	<0.001
ALP (U) [median (IQR)]	104.50 (80.88, 137.25)	113.50 (69.50, 152.63)	<0.001
CK (U/L) [median (IQR)]	511.50 (123.25, 1,259.75)	321.50 (200.29, 568.25)	0.004
CKMB (ng/ml) [median (IQR)]	12.53 (7.48, 221.75)	18.58 (7.49, 49.10)	<0.001
BNP (pg/ml) [median (IQR)]	378.85 (286.17, 1,109.50)	1,301.50 (195.25, 3,498.50)	0.040
K^+^ (mmol/L) [mean (SD)]	4.00 (0.11)	4.04 (0.76)	<0.001
Na^+^ (mmol/L) [mean (SD)]	139.43 (1.27)	139.64 (0.71)	<0.001
Ca^2+^ (mmol/L) [mean (SD)]	7.83 (0.15)	7.91 (0.16)	<0.001
Cl^−^ (mmol/L) [mean (SD)]	104.99 (1.58)	105.02 (1.42)	<0.001
Anion gap (mmol/L) [median (IQR)]	11.00 (8.25, 15.75)	11.58 (7.63, 13.81)	<0.001
PH [mean (SD)]	7.37 (0.16)	7.33 (0.02)	<0.001
HCO_3_^−^ (mmol/L) [mean (SD)]	25.58 (0.77)	24.38 (1.00)	<0.001
Lactate (mmol/L) [median (IQR)]	2.41 (1.48, 3.40)	2.43 (1.70, 3.60)	<0.001
Glucose (mg/dl) [mean (SD)]	137.68 (7.12)	149.14 (8.97)	<0.001
Treatment information, *n* (%)			
Ventilation, *n* (%)	921 (63.96)	928 (80.28)	<0.001
Epinephrine, *n* (%)	139 (9.65)	190 (16.44)	<0.001
Dopamine, *n* (%)	116 (8.06)	138 (11.94)	<0.001
Vasopressin, *n* (%)	116 (8.06)	202 (17.474)	<0.001
Marking systems			
GCS [median (IQR)]	9.57 (6.00, 13.90)	4.00 (3.00, 7.00)	<0.001
CCI [median (IQR)]	2.00 (1.00, 3.00)	2.50 (1.00, 4.50)	0.002

### 3.2 Data processing

**Figure 2:**
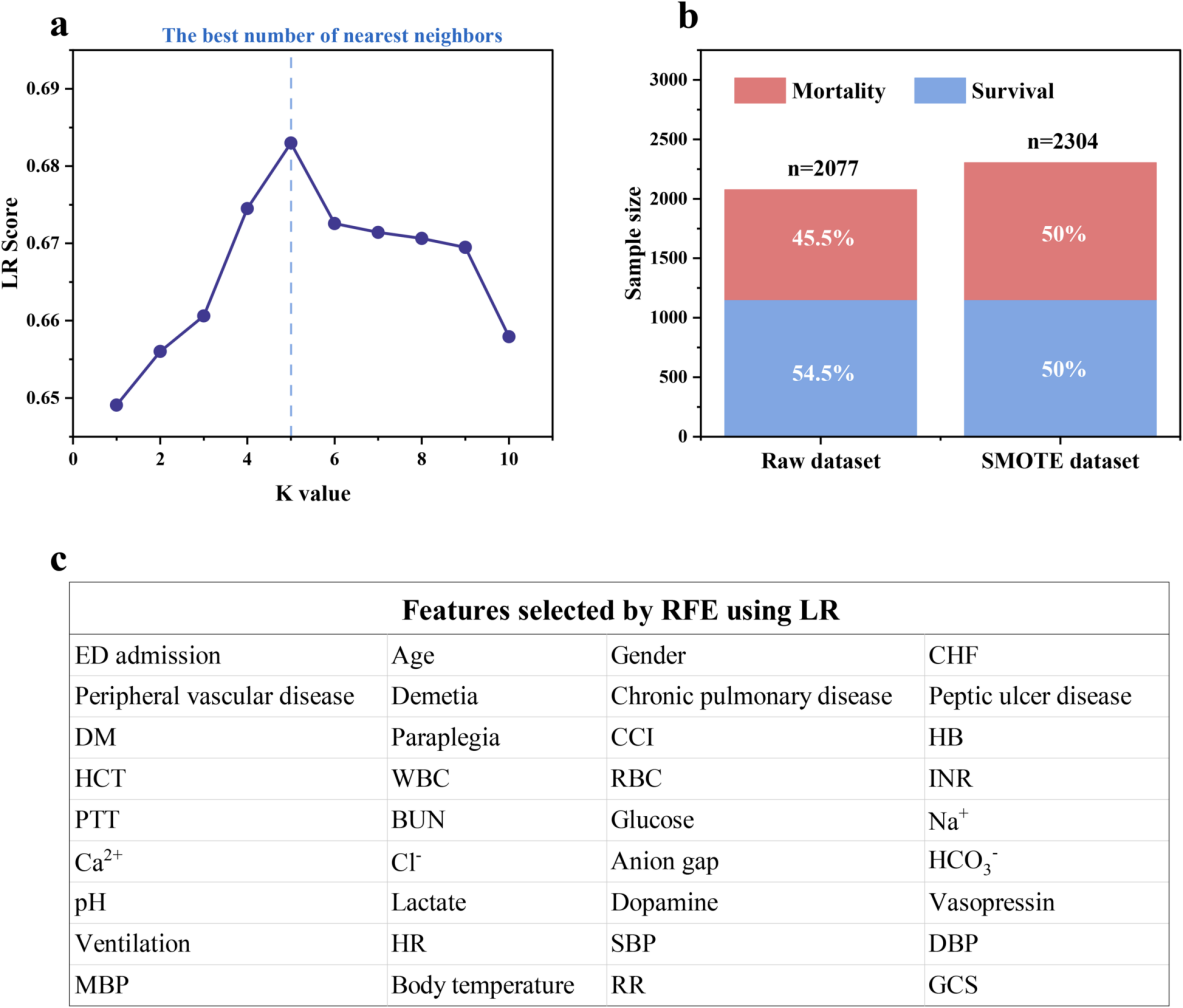
**(a)** The missing value imputation using KNN under different *K* values; **(b)** training cohort balancing using SMOTE; **(c)** feature selection using RFE based on LR.

KNN was used to impute missing values, and the details were described in Supplementary Text S1. Logistic regression (LR) was established to evaluate the effect of KNN imputer with different *K* values to achieve the best imputation result and avoid potential bias on candidate models. Figure 2a illustrates that the accuracy of LR peaked at 0.683 when the *K* value reached 5 and then decreased. Hence, the *K* value was set to 5 to impute missing values. Furthermore, in the training cohort, the in-hospital death rate of IHCA patients was 45.5% (1,152 survivors and 925 non-survivors), which may impede the prediction performance of ML models due to class imbalance (22). Figure 2b indicates that SMOTE could effectively balance the raw dataset by synthesizing new samples from the minority class in eICU-CRD. To avoid potential bias of the selected features on candidate models, LR was employed for recursive feature elimination (RFE) to initially screen for significant clinical features. The results demonstrate that RFE using LR decreased the original 54 features into a subset of 36 features, achieving the optimal combination of features (Figure 2c).

### 3.3 Model performance comparison

**Figure 3:**
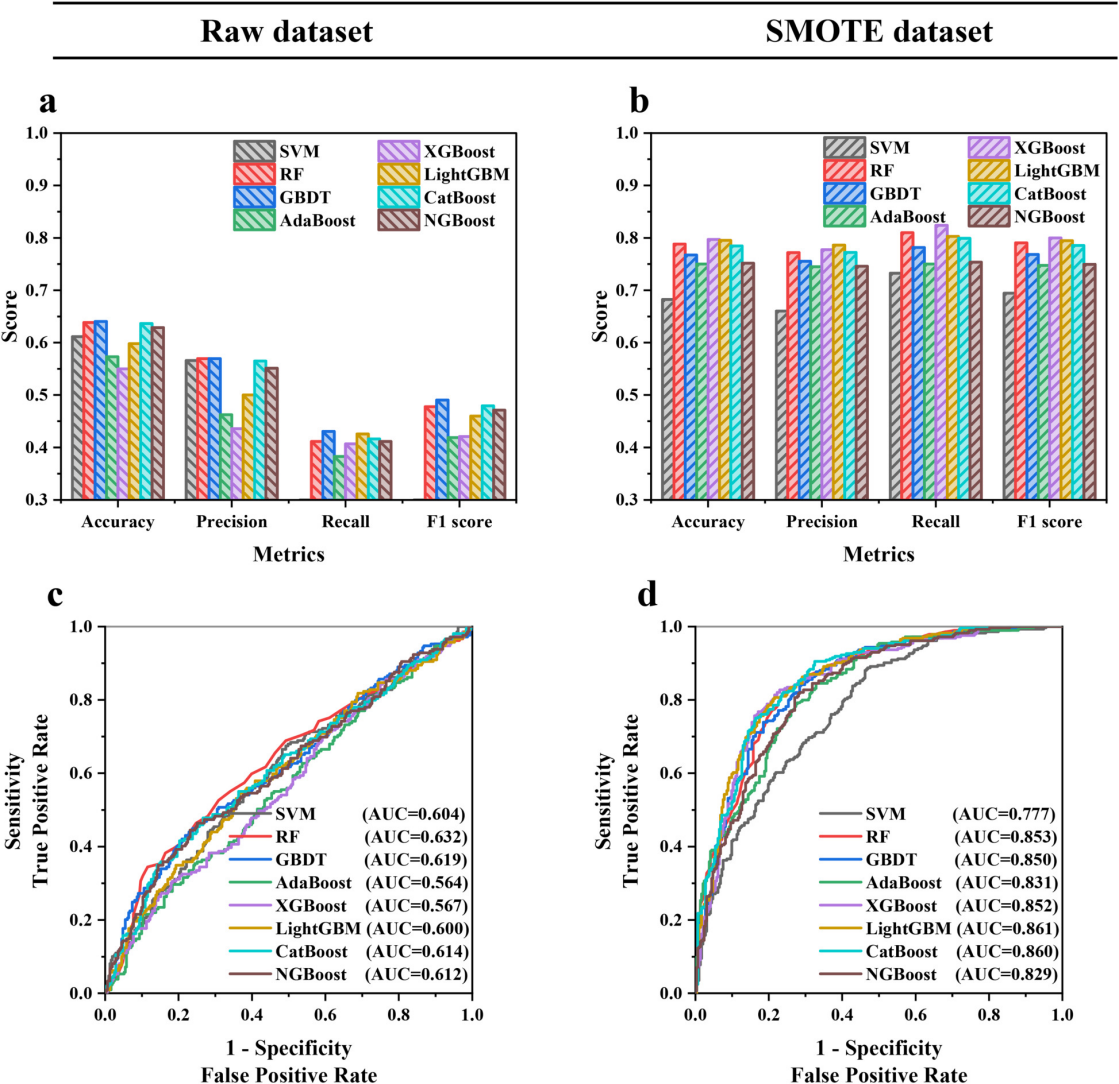
**(a,b)** Accuracy, precision, recall, F1 score and **(c,d)** AUC in the internal validation cohort of eight ML models trained with the raw dataset and SMOTE dataset.

The hyperparameters were tuned using grid search to reach the best performance of each ML model, and the training results are shown in Supplementary Figure S1. Figure 3 indicates that the balanced sample generated by SMOTE consistently improved the model performance in the internal validation cohort. The average accuracy, precision, recall, F1 score, and AUC of ML models were increased by 25.4%, 42.5%, 144.7%, 77.8%, and 34.5%, respectively, showing that SMOTE could enhance the prediction capability of models, which might be due to the advantages of SMOTE in balancing the training cohort, introducing diversity and reducing the risk of overfitting (30). Furthermore, Supplementary Table S3 indicates that XGBoost and LightGBM had the best performance, with the highest accuracy, recall, and F1 score of 0.797, 0.824, and 0.800 in XGBoost and the highest precision and AUC of 0.786 and 0.861 in LightGBM, respectively. Therefore, XGBoost and LightGBM developed with SMOTE were used for ensemble modeling to obtain better prediction performance and generalization capability.

### 3.4 Soft voting ensemble modeling

**Figure 4:**
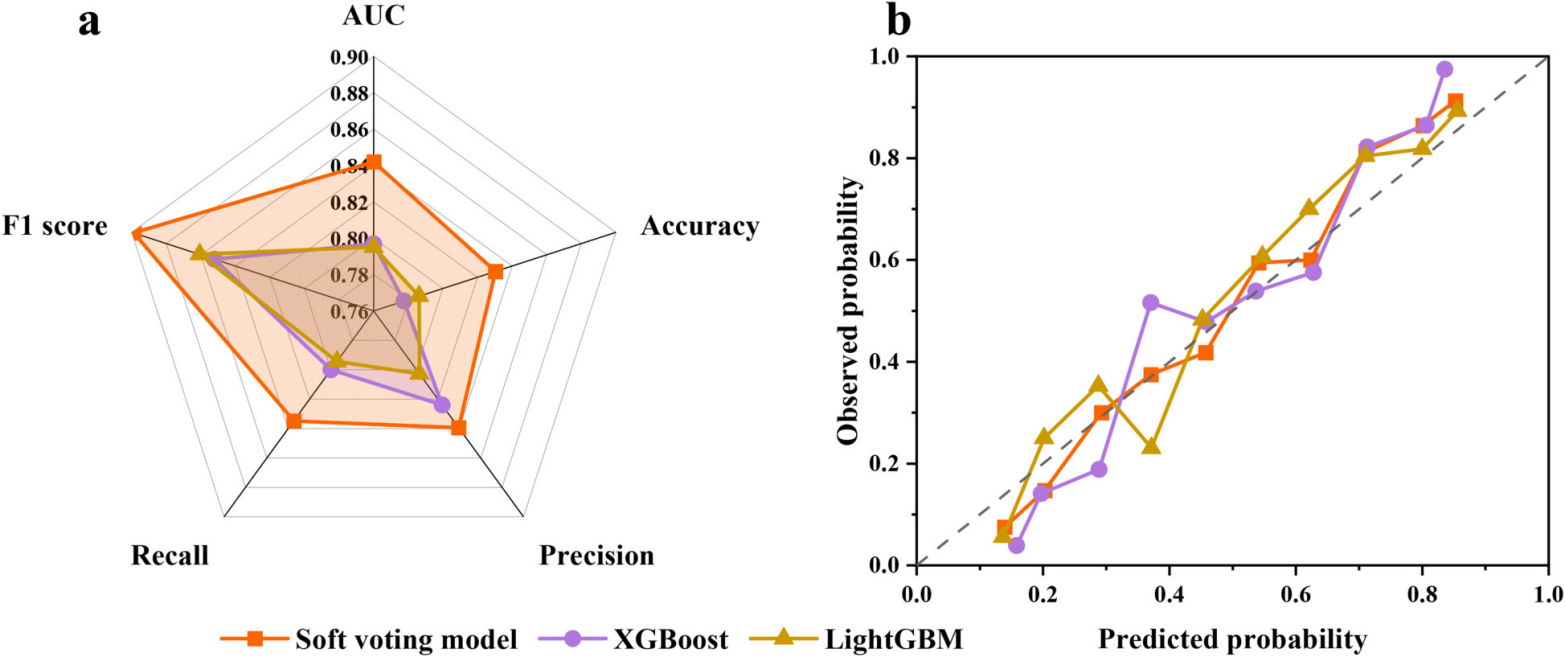
**(a)** Prediction performance of the EL model in the internal validation cohort. **(b)** The calibration plot illustrates the agreement between predicted probabilities and observed probabilities of survival.

The training results and prediction performance of the EL model are shown in Supplementary Figure S2 and Figure 4a, respectively. The accuracy, precision, recall, F1 score, and AUC of the EL model all reach 1 in the training cohort. In the internal validation cohort, the accuracy, precision, recall, F1 score, and AUC of the EL model exceed XGBoost and LightGBM, reaching 0.842 (vs. 0.797 in XGBoost), 0.830 (vs. 0.786 in LightGBM), 0.839 (vs. 0.824 in XGBoost), 0.835 (vs. 0.800 in XGBoost), and 0.898 (vs. 0.861 in LightGBM), respectively. In addition, the EL model showed better calibration across the spectrum of survival probabilities, with the lowest Brier score of 0.137 (Figure 4b; Supplementary Table S3).

### 3.5 Model interpretation

**Figure 5:**
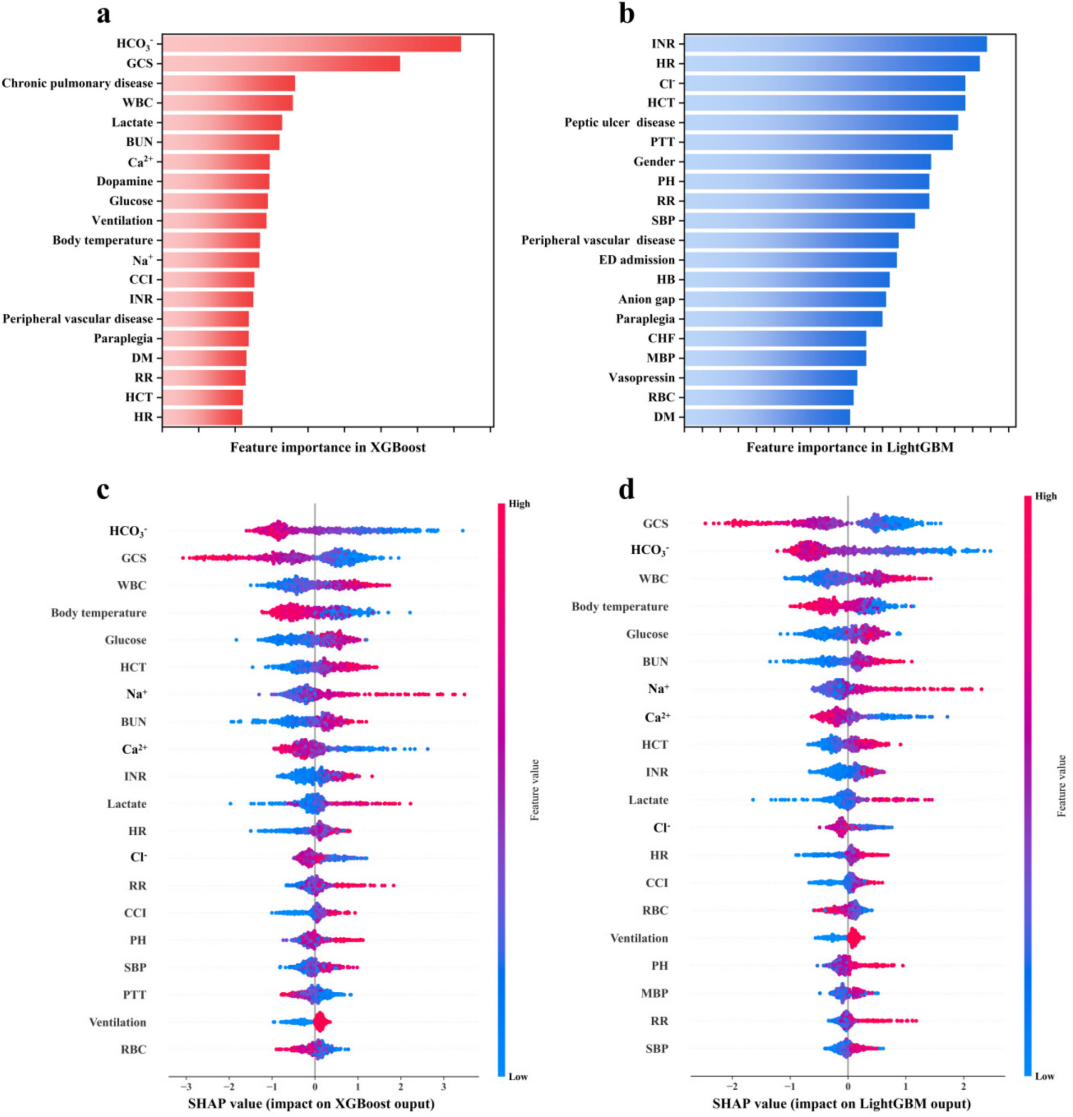
Feature importance ranking derived by the **(a,b)** Gini impurity and **(c,d)** SHAP method.

The contribution of each feature to the death risk prediction in LightGBM and XGBoost was evaluated using the Gini impurity (Figures 5a,c) and SHAP method (Figures 5c,d). For the SHAP method, on the vertical axis, each feature was ranked according to the mean |SHAP| value of each feature across all samples, which indicates the global importance of each feature; on the horizontal axis, the SHAP value for each sample is shown, which represent the distribution of the influence of features on the model output. The colors denote feature values (red for high and blue for low), which illustrate the impact of feature variation on outcomes. Figure 5 indicates that INR, HR, Cl^−^, HCT, PH, RR, SBP, MBP, and RBC were considered essential for LightGBM, while HCO_3_^−^, GCS, WBC, lactate, BUN, Ca^2+^, glucose, ventilation, body temperature, Na^+^, CCI, INR, HCT, and HR were found to be critical for XGBoost.

### 3.6 Input feature optimization

**Figure 6:**
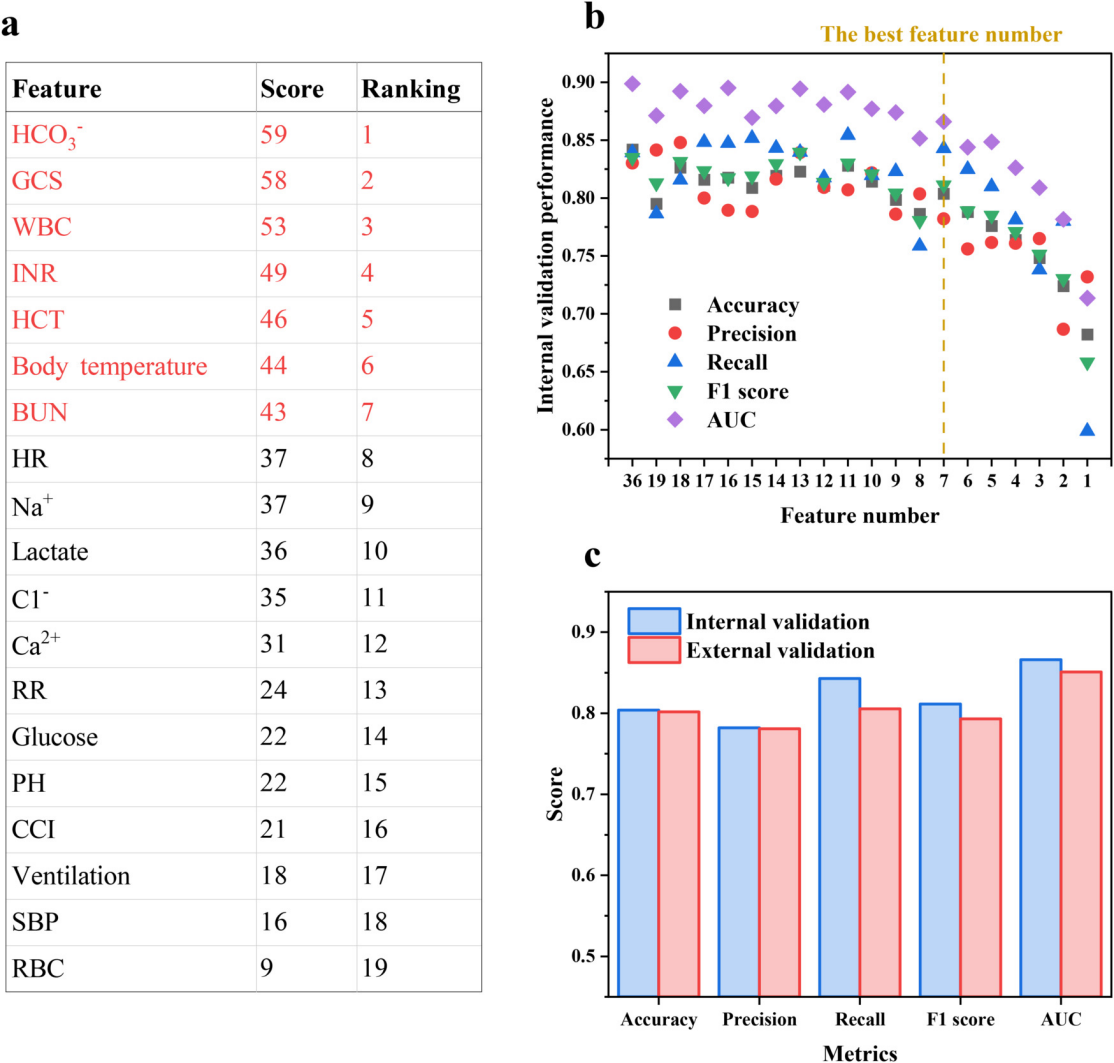
**(a)** The composite feature importance score ranking and **(b)** accuracy, precision, recall, F1 score and AUC of the EL model with decreasing feature number based on the ranking. **(c)** Prediction performance of the EL model in the external validation cohort.

Given that all basic models in the EL model were tree-based, the Gini impurity and SHAP method were used together to reveal the final feature importance ranking to avoid individual bias. Features recurring in Figure 5 were included for feature importance scoring, and the scoring rules are described in Supplementary Text S2 and Supplementary Table S4. Features that obtain scores are deemed by the EL model to be important for predicting IHCA death risk, and the composite score ranking is shown in Figure 6a. Next, each feature was eliminated sequentially starting from the least contributing feature to optimize input features and reduce model complexity. Fewer features without compromising model performance imply faster response times, which can enhance the use value of the prediction model.

Figure 6b demonstrates that there is no obvious performance decline in the internal validation cohort with the top seven features retained, with accuracy, precision, recall, F1 score, and AUC of 0.804, 0.782, 0.842, 0.811, and 866, respectively. As an example of accuracy, the performance decreased by 6.60% when using the top eight features, while the accuracy decreased by 4.54% for the top seven features and by 6.39% when further reduced to six features. Moreover, no significant multicollinearity among the seven features was observed via Pearson correlation coefficients (<|0.5|), confirming that these features are independent in their linear relationships and ensuring that the contribution of each feature to mortality risk is accurately quantified (Supplementary Figure S3). Thus, employing clinical features including HCO_3_^−^, GCS, WBC, INR, HCT, body temperature, and BUN as inputs allowed the EL model possible to predict with high precision using as few features as possible, which could help physicians make fast and accurate decisions in time-critical and complex emergencies to gain valuable time to improve the curability and prognostic for IHCA patients.

### 3.7 External validation

The MIMIC-IV database was used for external validation, and the results are shown in Figure 6c. In the external validation, the final model gave a similar performance to that in the internal validation, with accuracy, precision, recall, F1 score, and AUC of 0.802, 0.781, 0.805, 0.793, and 0.851, respectively, indicating that the final model showed strong capability and robustness in prediction and extrapolation. Moreover, the consistency of EL model performance across cohorts further emphasizes the positive impact of SMOTE on model performance, indicating that the synthetic samples generated by SMOTE did not introduce significant biases.

### 3.8 Web application

**Figure 7:**
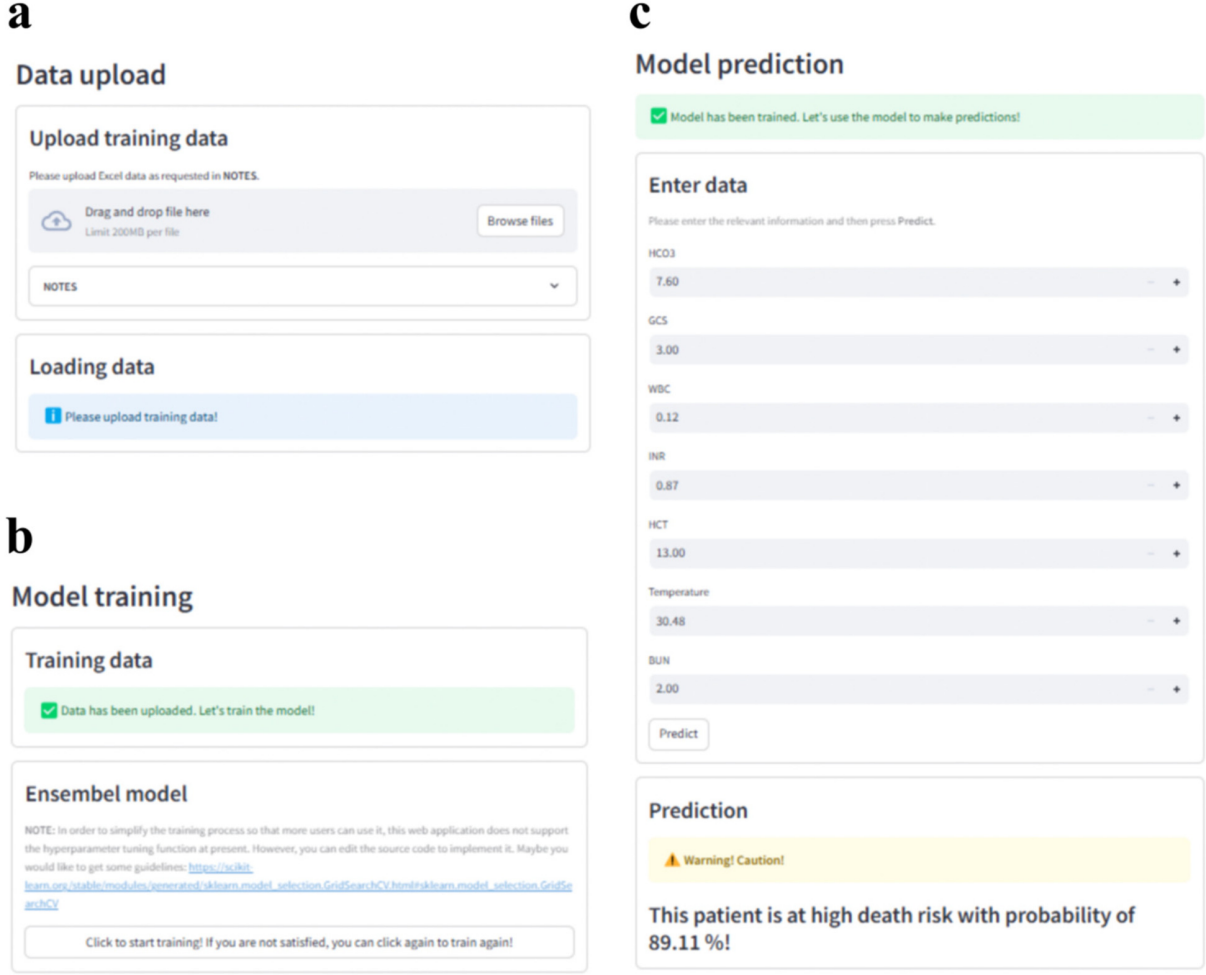
Web application with **(a)** data upload, **(b)** model training and **(c)** model prediction modules for clinical utility. Screenhots from: https://elm4ihca.streamlit.app/.

The final prediction model was implemented into the web application to facilitate its utility in clinical scenarios, as shown in Figure 7. When the actual values of the seven features required for the model are entered, this application will automatically predict the death risk for an individual CA patient. The web application is available at https://elm4ihca.streamlit.app/, which follows the security framework of Streamlit Community Cloud. Note that the application is for research and providing support for clinicians, not for direct patient care.

## 4 Discussion

IHCA is a major adverse event with a high death risk if not treated appropriately (39, 40). It is reported that the in-hospital death rate for patients who developed IHCA was 81.46% compared with 23.83% among patients who did not develop IHCA (41). Providing excellent clinical advice is important for doctors to assess CA severity and plan more appropriate treatment based on patient information, thereby reducing suffering and costs by avoiding futile rescue efforts. With respect to target temperature management (TTM), Callaway et al. (42) indicated that choosing TTM at 33°C was associated with better outcomes than TTM at 36°C for patients with severe post-CA illness, but TTM at 36°C was associated with better survival in mild- to moderate-severity illness. Therefore, our study developed a clinical prediction model with good performance to predict the death risk of IHCA patients, which utilized clinical features that can be collected in most clinical settings.

The proposed EL model could make prediction with high precision in internal and external validation cohorts using only seven clinical features, which is driven by the more comprehensive feature importance analysis and the fact that the EL model could effectively correct overfitting and underfitting tendencies inherent in single models to enhance prediction performance and generalization capability. More importantly, the seven clinical features used to assess IHCA death risk were related to the basic vital signs, neurologic function scores, and laboratory tests of patients, including body temperature, GCS, HCT, WBC, INR, BUN, and HCO_3_^−^, which are routine examinations when a patient is first admitted to the hospital. Thus, these input features can be rapidly obtained in most clinical settings when patients experience CA, implying our model is more practical. The developed web application employed the final EL model with the seven clinical features to calculate death risk for IHCA patients.

Furthermore, the findings in model interpretation ensured the consistency of our model with fundamental domain knowledge and clinical experience. HCO_3_^−^ was proven to be associated with death risk of CA patients in a multicenter prospective study (43). During the ischemic phase post-CA, anaerobic glycolysis will lead to lactic acid accumulation and subsequent tissue acidosis (44). Previous studies also found that the lower the GCS score, the higher the death risk (45, 46). In addition, some studies have pointed out that the systemic inflammatory response plays an important role in the pathophysiological development of early post-CA syndrome and affects the prognosis of IHCA patients, while WBC is the early determinant of the development of the systemic inflammatory response (47, 48). During ischemia and reperfusion in CPR, neutrophils will be activated to release inflammatory mediators, such as elastase and heparin-binding protein, which can lead to inflammatory tissue damage and therefore affect the prognosis of patients (49, 50). Abnormity in the coagulation–fibrinolysis system is an important pathophysiological feature of CA patients (51, 52), and thus INR was proven to be an independent risk factor for predicting the in-hospital death rate in CA patients (53, 54). Furthermore, den Hartog et al. (55) found that initial hypothermia in CA patients was associated with poor prognosis and poor neurologic recovery.

In terms of model performance and practicality, our model has enhanced prediction performance and generalization capability, simple operation, and fast calculation, which makes it more suitable for urgent clinical scenarios than a few similar studies. Chen et al. (56) developed a nomogram to predict in-hospital death risk for CA patients based on the MIMIC-IV database, whereas our study used two large databases, including MIMIC-IV and eICU-CRD, which have extensive data to demonstrate a more comprehensive patient condition. Bi et al. (57) developed an in-hospital death risk prediction model using 69 clinical features based on ML methods, which showed good prediction performance with an AUC of 0.86. However, the usefulness of the prediction model was limited since it is improbable that any clinician will be able to enter data on 69 clinical features during ongoing CPR. Moreover, DL has advanced healthcare prediction in domains such as medical imaging and regular time-series analysis, but its utility in tabular data is limited, despite tabular data being the most common data type in electronic medical records (EMRs) (33, 34). In contrast, the proposed parsimonious EL model could directly model non-linear feature interactions and used only seven clinical features to achieve AUC of 0.84 in internal validation and AUC of 0.81 in external validation, which yielded a clinically actionable tool with interpretable feature contributions, millisecond inference speed, and robust performance that are paramount for rapid and explainable clinical decisions. Meanwhile, Supplementary Table S5 revealed no significant differences in the seven key features between misclassified and overall cases, indicating the model errors are not driven by feature distribution and further demonstrating the robustness of feature selection.

The error analysis also provides the motivation to continue improving the EL model in future work. The EL model requires a prospective study to further assess its impact on clinical decisions in actual clinical settings and to identify areas where the model fails or underperforms. In addition, there is a risk of selection bias due to the fact that MIMIC-IV database and eICU-CRD may not represent all patients with CA in the ICU. Future research should use data from more diverse sources to avoid this risk. As a retrospective study, it faces inconsistent quality and completeness of recorded clinical features; thus, more advanced methods or prospective data collection are needed. Meanwhile, unmeasured confounding factors like CPR quality and genetic factors, which can influence in-hospital mortality risk, were not considered. Future studies should incorporate them to enhance the accuracy of risk prediction models. Although our EL model has achieved high performance in predicting the mortality of CA patients in the ICU, there are still some errors. Therefore, clinicians should always combine their clinical judgment with the model prediction rather than relying solely on the model to maximize patient protection.

## 5 Conclusion

Overall, the EL model outperformed all single ML models, including SVM, RF, AdaBoost, GBDT, XGBoost, LightGBM, CatBoost, and NGBoost, in predicting the death risk of IHCA patients. HCO_3_^−^, GCS, WBC, INR, HCT, body temperature, and BUN were considered as the most important input features of the EL model when a patient experiences CA, with no significant impact on model performance if only retaining them. Therefore, the parsimonious EL model can reliably and rapidly estimate the death risk of IHCA patients, which can provide clinicians with critical information in urgent clinical settings.

## Data Availability

Publicly available datasets were analyzed in this study. This data can be found here: https://physionet.org/content/mimiciv/2.2/ https://eicu-crd.mit.edu.

## References

[1] Dukes K Bunch JL Chan PS Guetterman TC Lehrich JL Trumpower B Assessment of rapid response teams at top-performing hospitals for in-hospital cardiac arrest. JAMA Intern Med. (2019) 179(10):1398–405. 10.1001/jamainternmed.2019.2420 31355875 PMC6664378

[2] Chan ML Spertus JA Tang F Jayaram N Chan PS. Missed opportunities in use of medical emergency teams prior to in-hospital cardiac arrest. Am Heart J. (2016) 177:87–95. 10.1016/j.ahj.2016.04.014 27297853 PMC4908832

[3] Holmberg MJ Ross CE Fitzmaurice GM Chan PS Duval-Arnould J Grossestreuer AV Annual incidence of adult and pediatric in-hospital cardiac arrest in the United States. Circ Cardiovasc Qual Outcomes. (2019) 12(7):e005580. 10.1161/CIRCOUTCOMES.119.005580 31545574 PMC6758564

[4] Go AS Mozaffarian D Roger VL Benjamin EJ Berry JD Blaha MJ Heart disease and stroke statistics–2014 update: a report from the American Heart Association. Circulation. (2014) 129(3):e28–292. 10.1161/01.cir.0000441139.02102.80 24352519 PMC5408159

[5] Jakkula P Skrifvars MB Pettilä V Hästbacka J Reinikainen M. NSE concentrations and haemolysis after cardiac arrest. Intensive Care Med. (2019) 45(5):741–2. 10.1007/s00134-019-05547-8 30758520

[6] Hoiland RL Rikhraj KJK Thiara S Fordyce C Kramer AH Skrifvars MB Neurologic prognostication after cardiac arrest using brain biomarkers: a systematic review and meta-analysis. JAMA Neurol. (2022) 79(4):390–8. 10.1001/jamaneurol.2021.5598 35226054 PMC8886448

[7] Drabek T Janata A Wilson CD Stezoski J Janesko-Feldman K Tisherman SA Minocycline attenuates brain tissue levels of TNF-α produced by neurons after prolonged hypothermic cardiac arrest in rats. Resuscitation. (2014) 85(2):284–91. 10.1016/j.resuscitation.2013.10.015 24513126 PMC3952024

[8] Meyer MAS Wiberg S Grand J Meyer ASP Obling LER Frydland M Treatment effects of interleukin-6 receptor antibodies for modulating the systemic inflammatory response after out-of-hospital cardiac arrest (the IMICA trial): a double-blinded, placebo-controlled, single-center, randomized, clinical trial. Circulation. (2021) 143(19):1841–51. 10.1161/circulationaha.120.053318 33745292 PMC8104015

[9] Annborn M Dankiewicz J Erlinge D Hertel S Rundgren M Smith JG Procalcitonin after cardiac arrest - an indicator of severity of illness, ischemia-reperfusion injury and outcome. Resuscitation. (2013) 84(6):782–7. 10.1016/j.resuscitation.2013.01.004 23313427

[10] Matsuda J Kato S Yano H Nitta G Kono T Ikenouchi T The sequential organ failure assessment (SOFA) score predicts mortality and neurological outcome in patients with post-cardiac arrest syndrome. J Cardiol. (2020) 76(3):295–302. 10.1016/j.jjcc.2020.03.007 32305260

[11] Sun Y He Z Ren J Wu Y. Prediction model of in-hospital mortality in intensive care unit patients with cardiac arrest: a retrospective analysis of MIMIC-IV database based on machine learning. BMC Anesthesiol. (2023) 23(1):178. 10.1186/s12871-023-02138-5 37231340 PMC10210383

[12] Shang LX Zhou XH Zhang JH Zhang WH TuEr-Hong ZL Zhao Y Establishment of a predictive model for inpatient sudden cardiac death in a Chinese cardiac department population: a retrospective study. Chin Med J. (2019) 132(1):17–24. 10.1097/cm9.0000000000000010 30628955 PMC6629305

[13] Wang D Xiang H. Composite control of post-chlorine dosage during drinking water treatment. IEEE Access. (2019) 7:27893–8. 10.1109/ACCESS.2019.2901059

[14] Corominas L Garrido-Baserba M Villez K Olsson G Cortés U Poch M. Transforming data into knowledge for improved wastewater treatment operation: a critical review of techniques. Environ Model Softw. (2018) 106:89–103. 10.1016/j.envsoft.2017.11.023

[15] Zhong S Zhang K Bagheri M Burken JG Gu A Li B Machine learning: new ideas and tools in environmental science and engineering. Environ Sci Technol. (2021) 55(19):12741–54. 10.1021/acs.est.1c01339 34403250

[16] Li L Rong S Wang R Yu S. Recent advances in artificial intelligence and machine learning for nonlinear relationship analysis and process control in drinking water treatment: a review. Chem Eng J. (2021) 405:126673. 10.1016/j.cej.2020.126673

[17] Li J Gong M Joshi Y Sun L Huang L Fan R Machine learning prediction model for acute renal failure after acute aortic syndrome surgery. Front Med. (2022) 8:728521. 10.3389/fmed.2021.728521 PMC8801502 35111767

[18] Ge W Huh J-W Park YR Lee J-H Kim Y-H Zhou G Using deep learning with attention mechanism for identification of novel temporal data patterns for prediction of ICU mortality. Inform Med Unlocked. (2022) 29:100875. 10.1016/j.imu.2022.100875

[19] Telangore H Azad V Sharma M Bhurane A Tan RS Acharya UR. Early prediction of sudden cardiac death using multimodal fusion of ECG features extracted from Hilbert–Huang and wavelet transforms with explainable vision transformer and CNN models. Comput Methods Programs Biomed. (2024) 257:108455. 10.1016/j.cmpb.2024.108455 39447439

[20] Li B Liu L Ma R Guo L Jiang J Li K Siamese based few-shot learning lightweight transformer model for coagulant and disinfectant dosage simultaneous regulation. Chem Eng J. (2024) 499:156025. 10.1016/j.cej.2024.156025

[21] Xu J Zhang X Zhao C Geng Z Feng Y Miao K Improving fine-grained image classification with multimodal information. IEEE Trans Multimedia. (2024) 26:2082–95. 10.1109/TMM.2023.3291819

[22] Hessulf F Bhatt DL Engdahl J Lundgren P Omerovic E Rawshani A Predicting survival and neurological outcome in out-of-hospital cardiac arrest using machine learning: the SCARS model. EBioMedicine. (2023) 89:104464. 10.1016/j.ebiom.2023.104464 36773348 PMC9945645

[23] Zhang C Ma Y. Ensemble Machine Learning: Methods and Applications. New York, NY: Springer (2012). p. 1–329. https://link.springer.com/book/10.1007/978-1-4419-9326-7#publish-with-us

[24] Li B Liu L Xu Z Li K. Optimizing carbon source addition to control surplus sludge yield via machine learning-based interpretable ensemble model. Environ Res. (2025) 267:120653. 10.1016/j.envres.2024.120653 39701344

[25] Jiang D Lei T Wang Z Shen C Cao D Hou T. ADMET evaluation in drug discovery. 20. Prediction of breast cancer resistance protein inhibition through machine learning. J Cheminform. (2020) 12(1):16. 10.1186/s13321-020-00421-y 33430990 PMC7059329

[26] Xu X Yu Z Ge Z Chow EPF Bao Y Ong JJ Web-based risk prediction tool for an individual’s risk of HIV and sexually transmitted infections using machine learning algorithms: development and external validation study. J Med Internet Res. (2022) 24(8):e37850. 10.2196/37850 36006685 PMC9459839

[27] Shahhosseini M Hu G Archontoulis SV. Forecasting corn yield with machine learning ensembles [methods]. Front Plant Sci. (2020) 11:1120. 10.3389/fpls.2020.01120 32849688 PMC7411227

[28] Salih AM Raisi-Estabragh Z Galazzo IB Radeva P Petersen SE Lekadir K A perspective on explainable artificial intelligence methods: SHAP and LIME. Adv Intell Syst. (2025) 7(1):2400304. 10.1002/aisy.202400304

[29] Merrick L Taly A. The explanation game: explaining machine learning models using Shapley values. In: Holzinger A Kieseberg P Tjoa AM Weippl E, editors. Machine Learning and Knowledge Extraction. Cham: Springer International Publishing (2020). p. 17–38.

[30] Chawla N Bowyer K Hall L Kegelmeyer W. SMOTE: synthetic minority over-sampling technique. J Artif Intell Res. (2002) 16:321–57. 10.1613/jair.953

[31] Xu H Li C Shi T. Is the z-score standardized RSEI suitable for time-series ecological change detection? Comment on Zheng et al. (2022). Sci Total Environ. (2022) 853:158582. 10.1016/j.scitotenv.2022.158582 36089031

[32] Marateb HR Ziaie Nezhad F Mohebian MR Sami R Haghjooy Javanmard S Dehghan Niri F Automatic classification between COVID-19 and non-COVID-19 pneumonia using symptoms, comorbidities, and laboratory findings: the Khorshid COVID cohort study. Front Med. (2021) 8:768467. 10.3389/fmed.2021.768467 PMC8640954 34869483

[33] Hollmann N Müller S Eggensperger K Hutter F. TabPFN: a transformer that solves small tabular classification problems in a second. In: International Conference on Learning Representations; (2023).

[34] Hollmann N Müller S Purucker L Krishnakumar A Körfer M Hoo SB Accurate predictions on small data with a tabular foundation model. Nature. (2025) 637(8045):319–26. 10.1038/s41586-024-08328-6 39780007 PMC11711098

[35] Sundararajan M Najmi A. The many Shapley values for model explanation. In: Proceedings of the 37 International Conference on Machine Learning; (2020). p. 859.

[36] Zhang K Zhong S Zhang H. Predicting aqueous adsorption of organic compounds onto biochars, carbon nanotubes, granular activated carbons, and resins with machine learning. Environ Sci Technol. (2020) 54(11):7008–18. 10.1021/acs.est.0c02526 32383863

[37] Orozco JIJ Knijnenburg TA Manughian-Peter AO Salomon MP Barkhoudarian G Jalas JR Epigenetic profiling for the molecular classification of metastatic brain tumors. Nat Commun. (2018) 9(1):4627. 10.1038/s41467-018-06715-y 30401823 PMC6219520

[38] Yuan Y Zhang L Long Q Jiang H Li M. An accurate prediction model of digenic interaction for estimating pathogenic gene pairs of human diseases. Comput Struct Biotechnol J. (2022) 20:3639–52. 10.1016/j.csbj.2022.07.011 35891796 PMC9289819

[39] Abrams D MacLaren G Lorusso R Price S Yannopoulos D Vercaemst L Extracorporeal cardiopulmonary resuscitation in adults: evidence and implications. Intensive Care Med. (2022) 48(1):1–15. 10.1007/s00134-021-06514-y 34505911 PMC8429884

[40] Gravesteijn BY Schluep M Disli M Garkhail P Dos Reis Miranda D Stolker RJ Neurological outcome after extracorporeal cardiopulmonary resuscitation for in-hospital cardiac arrest: a systematic review and meta-analysis. Crit Care. (2020) 24(1):505. 10.1186/s13054-020-03201-0 32807207 PMC7430015

[41] Duazo C Hsiung JC Qian F Sherrod CF Ling DA Wu IJ In-hospital cardiac arrest in patients with sepsis: a national cohort study. Front Med. (2021) 8:731266. 10.3389/fmed.2021.731266 PMC8553946 34722572

[42] Callaway CW Coppler PJ Faro J Puyana JS Solanki P Dezfulian C Association of initial illness severity and outcomes after cardiac arrest with targeted temperature management at 36°C or 33°C. JAMA Netw Open. (2020) 3(7):e208215. 10.1001/jamanetworkopen.2020.8215 32701158 PMC7378753

[43] Kim HC Yoo J-W Lim SY Suh GY Koh SO Na S Mortality after in-hospital cardiopulmonary resuscitation: multicenter analysis in Korea. J Crit Care. (2013) 28(6):942–6. 10.1016/j.jcrc.2013.07.048 23937967

[44] Ushay HM Notterman DA. Pharmacology of pediatric resuscitation. Pediatr Clin North Am. (1997) 44(1):207–33. 10.1016/s0031-3955(05)70470-3 9057791

[45] Imamura S Miyata M Tagata K Yokomine T Ohmure K Kawasoe M Prognostic predictors in patients with cardiopulmonary arrest: a novel equation for evaluating the 30-day mortality. J Cardiol. (2023) 82(2):146–52. 10.1016/j.jjcc.2023.01.006 36682713

[46] Nadolny K Bujak K Obremska M Zysko D Sterlinski M Szarpak L Glasgow Coma scale score of more than four on admission predicts in-hospital survival in patients after out-of-hospital cardiac arrest. Am J Emerg Med. (2021) 42:90–4. 10.1016/j.ajem.2021.01.018 33497899

[47] Adrie C Adib-Conquy M Laurent I Monchi M Vinsonneau C Fitting C Successful cardiopulmonary resuscitation after cardiac arrest as a “sepsis-like” syndrome. Circulation. (2002) 106(5):562–8. 10.1161/01.cir.0000023891.80661.ad 12147537

[48] Bro-Jeppesen J Kjaergaard J Wanscher M Nielsen N Friberg H Bjerre M Systemic inflammatory response and potential prognostic implications after out-of-hospital cardiac arrest: a substudy of the target temperature management trial. Crit Care Med. (2015) 43(6):1223–32. 10.1097/ccm.0000000000000937 25756419

[49] Gando S Tedo I. Increased neutrophil elastase release in patients with cardiopulmonary arrest: role of elastase inhibitor. Intensive Care Med. (1995) 21(8):636–40. 10.1007/bf01711540 8522666

[50] Huang L Peng J Wang X Li F. High platelet-lymphocyte ratio is a risk factor for 30-day mortality in in-hospital cardiac arrest patients: a case-control study. Expert Rev Clin Immunol. (2021) 17(11):1231–9. 10.1080/1744666X.2021.1994389 34696670

[51] Mizugaki A Wada T Tsuchida T Gando S. Association of histones with coagulofibrinolytic responses and organ dysfunction in adult post-cardiac arrest syndrome. Front Cardiovasc Med. (2022) 9:885406. 10.3389/fcvm.2022.885406 35837604 PMC9273886

[52] Neumar RW Nolan JP Adrie C Aibiki M Berg RA Böttiger BW Post-cardiac arrest syndrome: epidemiology, pathophysiology, treatment, and prognostication. A consensus statement from the International Liaison Committee on Resuscitation (American Heart Association, Australian and New Zealand Council on Resuscitation, European Resuscitation Council, Heart and Stroke Foundation of Canada, InterAmerican Heart Foundation, Resuscitation Council of Asia, and the Resuscitation Council of Southern Africa); the American Heart Association Emergency Cardiovascular Care Committee; the Council on Cardiovascular Surgery and Anesthesia; the Council on Cardiopulmonary, Perioperative, and Critical Care; the Council on Clinical Cardiology; and the Stroke Council. Circulation. (2008) 118(23):2452–83. 10.1161/circulationaha.108.190652 18948368

[53] Tang Y Sun J Yu Z Liang B Peng B Ma J Association between prothrombin time-international normalized ratio and prognosis of post-cardiac arrest patients: a retrospective cohort study. Front Public Health. (2023) 11:1112623. 10.3389/fpubh.2023.1112623 36741950 PMC9895096

[54] Fantz CR. Confirming point-of-care INR test results. J Am Med Assoc. (2020) 323(12):1190–1. 10.1001/jama.2020.0945 32207787

[55] den Hartog AW de Pont AC Robillard LB Binnekade JM Schultz MJ Horn J. Spontaneous hypothermia on intensive care unit admission is a predictor of unfavorable neurological outcome in patients after resuscitation: an observational cohort study. Crit Care. (2010) 14(3):R121. 10.1186/cc9077 20573203 PMC2911769

[56] Chen J Mei Z Wang Y Shou X Zeng R Chen Y A nomogram to predict in-hospital mortality in post-cardiac arrest patients: a retrospective cohort study. Pol Arch Intern Med. (2022) 133(1):16325. 10.20452/pamw.16325 35997470

[57] Bi S Chen S Li J Gu J. Machine learning-based prediction of in-hospital mortality for post cardiovascular surgery patients admitting to intensive care unit: a retrospective observational cohort study based on a large multi-center critical care database. Comput Methods Programs Biomed. (2022) 226:107115. 10.1016/j.cmpb.2022.107115 36126435

