# Supplementary material for: Prediction model of in-hospital mortality risk in intensive care unit patients with cardiac arrest: a multicenter retrospective cohort study based on an ensemble model
Source: Front Cardiovasc Med. 2025 May 20;12:1582636. doi: 10.3389/fcvm.2025.1582636 (PMC12131872; doi:10.3389/fcvm.2025.1582636)
Supplement: Supplementary file 1 [file Datasheet1.pdf]

Supplementary Material

Prediction model of in-hospital mortality risk in intensive care unit patients with cardiac arrest：a multicenter retrospective cohort study based on ensemble model

Li Liu[1], Wei-Wei Lai[1], Bo-Wen Li[2], Shu-Hang Wang[1], Mu-Ming Yu[1],Yan-Cun Liu[1*], Yan-Fen Chai[1*]

[1] Tianjin Medical University General Hospital, Tianjin 300052, China

[2] Nankai University, Tianjin 300350, China

Number of Pages: 13

Number of Figures: 3

Number of Tables: 5

Table S1. Baseline characteristics of the patients in mimic-iv database (n=1472).

Feature	Survival (n=778)	Death (n=694)	P value
**Patient characteristics**			
ED admission (n%)	372 (47.8)	382 (55.0)	0.006
Age (years old) (mean (SD))	71.27 (3.23)	69.56 (6.54)	0.074
Man(n%)	493 (63.4)	407 (58.6)	0.064
Height (cm) (mean (SD))	164.67 (3.92)	166.90 (1.63)	0.290
Weight (kg) (mean (SD))	79.71 (9.45)	89.03 (12.22)	0.072
**Comorbidities, n (%)**			
Myocardial infarct (n%)	246 (31.6)	194 (28.0)	0.125
CHF (n%)	366 (47.0)	274 (39.5)	0.003
Peripheral vascular disease (n%)	105 (13.5)	125 (18.0)	0.017
Cerebrovascular disease (n%)	102 (13.1)	134 (19.3)	0.001
Dementia (n%)	16 (2.1)	22 (3.2)	0.179
Immune system disease (n%)	24 (3.1)	27 (3.9)	0.399
Chronic pulmonary disease (n%)	199 (25.6)	188 (27.1)	0.511
Peptic ulcer disease (n%)	23 (3.0)	15 (2.2)	0.337
DM (n%)	264 (33.9)	259 (37.3)	0.175
Paraplegia (n%)	27 (3.5)	33 (4.8)	0.213
Renal disease (n%)	229 (29.4)	213 (30.7)	0.599
**Vital signs**			
HR (beats/minute) (mean (SD))	84.23 (5.12)	86.58 (5.55)	<0.001
SBP (mmHg) (mean (SD))	114.19 (5.55)	110.83 (4.21)	0.640
DBP (mmHg) (mean (SD))	59.17 (3.49)	59.80 (3.97)	0.689
MBP (mmHg) (mean (SD))	76.82 (3.34)	75.50 (2.60)	0.615
RR (beats/minute) (mean (SD))	20.21 (1.22)	23.30 (1.66)	<0.001
SPO2 (%) (mean (SD))	97.28 (0.49)	95.70 (0.90)	<0.001
Body temperature (℃) (mean (SD))	36.62 (0.12)	36.14 (0.44)	<0.001
**Laboratory tests**			
HB (g/dL) (mean (SD))	12.05 (1.21)	10.32 (0.51)	0.020
HCT (%) (mean (SD))	37.07 (3.77)	32.58 (1.84)	0.312
RBC (10^12/L) (mean (SD))	3.87 (0.31)	3.61 (0.22)	0.159
WBC (10^9/L) (mean (SD))	17.77 (2.19)	14.39 (0.90)	<0.001
Platelet (10^9/L) (mean (SD))	239.28 (36.81)	227.23 (49.05)	0.524

INR (mean (SD))	1.57 (0.31)	1.75 (0.16)	<0.001
PT (s) (mean (SD))	17.34 (3.55)	19.24 (1.63)	<0.001
PTT (s) (mean (SD))	46.71 (8.71)	46.32 (7.03)	0.611
Creatinine (mg/dL) (mean (SD))	1.53 (0.30)	1.80 (0.32)	<0.001
BUN (mg/dL) (mean (SD))	31.06 (5.49)	54.00 (11.54)	<0.001
AST (U/L) (median (IQR))	56.00 (35.80, 2137.5)	107.00 (43.36, 788.25)	<0.001
ALT (U/L) (median (IQR))	45.00 (24.00, 1399.50)	59.25 (29.38, 728.88)	<0.001
ALP (U) (mean (SD))	129.52 (33.13)	112.50 (16.52)	<0.001
CK (U/L) (median (IQR))	147.00 (60.17, 461.13)	207.25 (74.75, 424.50)	0.010
CKMB (ng/mL) (median (IQR))	4.33 (3.00, 12.25)	6.08 (2.63, 20.00)	<0.001
BNP (pg/mL) (mean (SD))	9006.22 (3479.46)	18674.60 (6506.71)	0.033
K^+ (mmol/L) (mean (SD))	4.41 (0.25)	4.40 (0.14)	0.427
Na^+ (mmol/L) (mean (SD))	137.79 (1.37)	138.51 (2.34)	<0.001
Ca^{2+} (mmol/L) (mean (SD))	7.98 (0.17)	8.26 (0.17)	0.108
Cl^- (mmol/L) (mean (SD))	103.21 (2.11)	102.44 (2.34)	0.123
Anion gap (mmol/L) (mean (SD))	16.90 (1.14)	17.95 (1.27)	<0.001
PH (mean (SD))	7.37 (0.02)	7.35 (0.02)	<0.001
HCO_3^- (mmol/L) (mean (SD))	21.81 (2.28)	21.37 (1.85)	<0.001
Lactate (mmol/L) (mean (SD))	2.29 (0.43)	3.29 (0.73)	<0.001
Glucose (mg/dL) (mean (SD))	168.95 (20.56)	180.37 (24.12)	<0.001
**Treatment information, n (%)**			
Ventilation (n, %)	719 (92.4)	651 (93.8)	0.295
Epinephrine (n, %)	107 (13.8)	120 (17.3)	0.061
Dopamine (n, %)	90 (11.6)	108 (15.6)	0.025
Vasopressin (n, %)	73 (9.4)	118 (17.0)	<0.001
**Marking systems**			
GCS (median (SD))	14.71 (0.15)	14.68 (0.20)	0.004
CCI (median (IQR))	5.00 (4.50, 8.50)	5.50 (4.00, 8.50)	<0.001

Table S2. Baseline characteristics of the patients between mimic-iv database and eICU-CRD database.

Feature	MIMIC-IV (n=1472)	eICU-CRD (n=2596)	P value
**Patient characteristics**			
ED admission (n%)	754 (51.2)	1141 (44.0)	<0.001
Age (years old) (median (IQR))	67.62 (56.43, 79.08)	65.00 (54.00, 75.00)	<0.001
Man(n%)	900 (61.1)	1533 (59.1)	0.192
Height (cm) (mean (SD))	170.11 (10.74)	170.04 (10.85)	0.856
Weight (kg) (median (IQR))	80.00 (68.90, 96.00)	82.50 (69.40, 100.00)	<0.001
**Comorbidities, n (%)**			
Myocardial infarct (n%)	440 (29.9)	316 (12.2)	<0.001
CHF (n%)	640 (43.5)	512 (19.7)	<0.001
Peripheral vascular disease (n%)	230 (15.6)	153 (5.9)	<0.001
Cerebrovascular disease (n%)	236 (16.0)	49 (1.9)	<0.001
Dementia (n%)	38 (2.6)	80 (3.1)	0.361
Immune system disease (n%)	51 (3.5)	54 (2.1)	0.007
Chronic pulmonary disease (n%)	387 (26.3)	419 (16.1)	<0.001
Peptic ulcer disease (n%)	38 (2.6)	38 (1.5)	0.011
DM (n%)	523 (35.5)	907 (34.9)	0.704
Paraplegia (n%)	60 (4.1)	218 (8.4)	<0.001
Renal disease (n%)	442 (30.0)	453 (17.4)	<0.001
**Vital signs**			
HR (beats/minute) (mean (SD))	83.60 (17.51)	82.30 (17.87)	0.027
SBP (mmHg) (mean (SD))	116.04 (16.00)	117.46 (18.36)	0.015
DBP (mmHg) (median (IQR))	61.96 (54.75, 68.71)	64.92 (58.48, 73.22)	<0.001
MBP (mmHg) (median (IQR))	76.92 (70.64, 84.11)	79.18 (72.15, 88.46)	<0.001
RR (beats/minute) (mean (SD))	20.31 (4.25)	20.01 (4.76)	0.045
SPO2 (%) (mean (SD))	97.16 (2.59)	97.35 (3.69)	0.077
Body temperature (℃) (median (IQR))	36.74 (36.41, 37.08)	36.52 (34.35, 37.03)	0.010
**Laboratory tests**			
HB (g/dL) (median (IQR))	11.00 (9.40, 12.95)	10.49 (9.09, 12.22)	<0.001
HCT (%) (median (IQR))	33.65 (28.70, 39.04)	32.10 (28.00, 36.67)	<0.001

RBC (10^12/L) (mean (SD))			<0.001
WBC (10^9/L) (median (IQR))	12.75 (9.20, 17.08)	12.30 (9.28, 16.17)	0.092
Platelet (10^9/L) (median (IQR))	183.67 (137.00, 245.42)	185.12 (138.54, 237.55)	0.611
INR (median (IQR))	1.30 (1.15, 1.60)	1.30 (1.13, 1.64)	0.985
PT (s) (median (IQR))	14.30 (12.70, 17.80)	15.46 (13.50, 19.10)	<0.001
PTT (s) (mean (SD))	45.25 (25.54)	44.76 (23.87)	0.603
Creatinine (mg/dL) (median (IQR))	1.20 (0.83, 1.94)	1.30 (0.86, 2.29)	0.001
BUN (mg/dL) (mean (SD))	30.10 (22.16)	31.09 (19.83)	0.178
AST (U/L) (median (IQR))	81.75 (39.00, 227.00)	124.50 (53.75, 339.00)	<0.001
ALT (U/L) (median (IQR))	54.00 (25.15, 162.25)	83.08 (37.00, 219.00)	<0.001
ALP (U) (mean (SD))	83.25 (61.00, 121.00)	89.00 (66.00, 126.00)	0.003
CK (U/L) (median (IQR))	277.50 (111.00, 816.13)	384.60 (182.00, 1064.50)	<0.001
CKMB (ng/mL) (median (IQR))	9.67 (4.33, 29.19)	13.35 (5.40, 42.31)	<0.001
BNP (pg/mL) (median (IQR))	4855.00 (1487.00, 15643.00)	893.5 (239.99, 2609.25)	<0.001
K^+ (mmol/L) (median (IQR))	4.17 (3.85, 4.57)	3.97 (3.73, 4.24)	<0.001
Na^+ (mmol/L) (mean (SD))	138.89 (4.87)	139.86 (4.61)	<0.001
Ca^{2+} (mmol/L) (median (IQR))	8.18 (7.70, 8.65)	8.06 (7.61, 8.48)	<0.001
Cl^- (mmol/L) (mean (SD))	104.63 (6.29)	105.21 (5.93)	0.007
Anion gap (mmol/L) (median (IQR))	21.00 (17.67, 23.50)	23.58 (20.64, 26.28)	<0.001
PH (median (IQR))	7.34 (7.28, 7.39)	7.34 (7.28, 7.40)	0.200
HCO_3^- (mmol/L) (mean (SD))	21.48 (4.55)	23.80 (4.43)	<0.001
Lactate (mmol/L) (median (IQR))	2.50 (1.67, 4.31)	2.52 (1.54, 4.25)	0.174
Glucose (mg/dL) (median (IQR))	160.71 (131.00, 214.10)	141.32 (121.08, 169.81)	<0.001
**Treatment information, n (%)**			
Ventilation (n, %)	1370 (93.1)	1849 (71.2)	<0.001
Epinephrine (n, %)	227 (15.4)	329 (12.7)	0.014
Dopamine (n, %)	198 (13.5)	254 (9.8)	<0.001
Vasopressin (n, %)	191 (13.0)	318 (12.2)	0.501
**Marking systems**			
GCS (median (IQR))	15.00 (14.60, 15.00)	7.11 (3.38, 10.78)	<0.001
CCI (median (IQR))	6.00 (4.00, 8.00)	2.00 (1.00, 4.00)	<0.001
**Death, n (%)**	694 (47.1)	1156 (44.5)	0.107

Text S1 Missing value imputation using KNN

The choice of K value can significantly influence the performance of KNN. The smaller K value implies consideration of fewer nearest neighbors to estimate missing values, which potentially makes the imputation results sensitive to individual outliers. The larger K value will use a more diverse set of samples to estimate missing values, which may introduce unrelated samples and thus affect the accuracy of imputation. In order to avoid potential bias of the selected K values on candidate models, logistic regression (LR) was employed to model datasets imputed using KNN with different K values and the accuracy of LR was calculated to evaluate the imputation effect, which ensured fairness to the eight ML models in the following modeling. The results are shown in **Fig. 2a**.

Raw dataset	SMOTE dataset

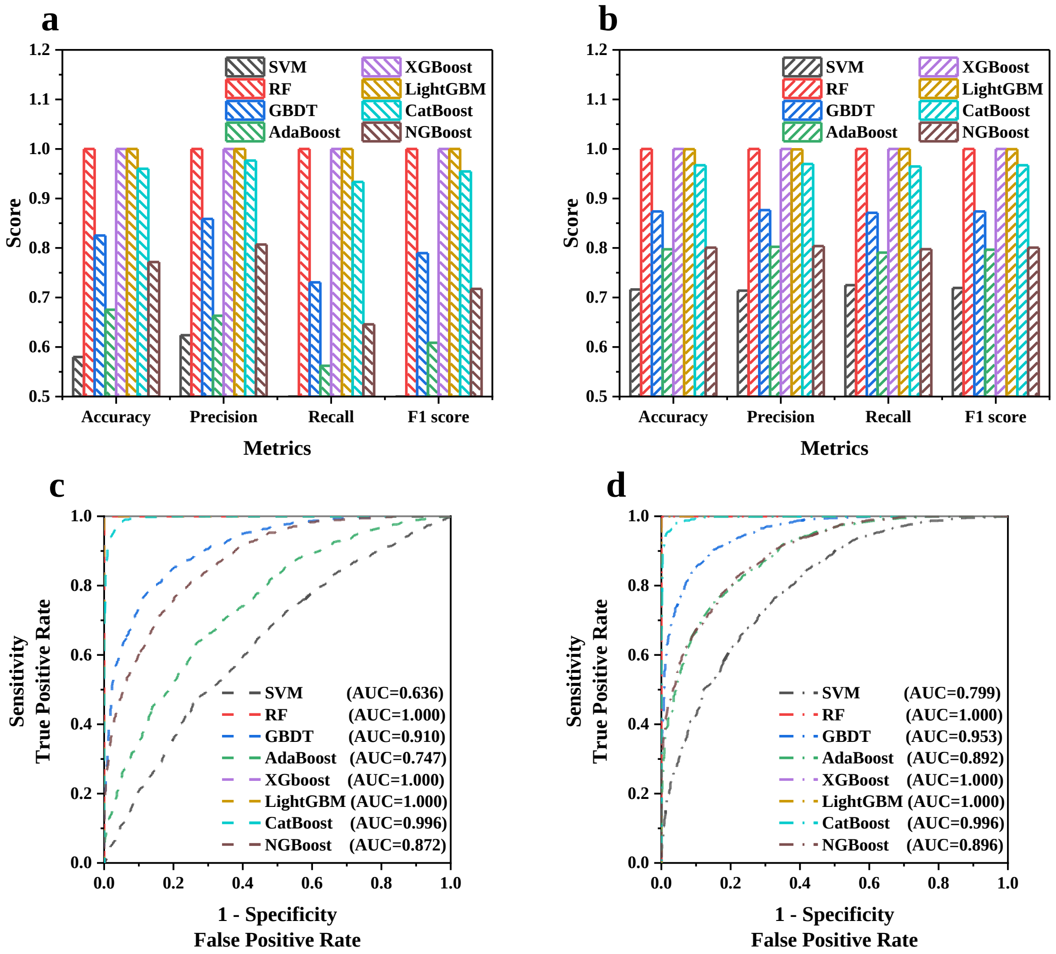

Fig. S1. Training results of eight ML models in the raw dataset and SMOTE dataset.

Table S3. Model performance in internal and external validation.

Model	Accuracy	Precision	Recall	F1 score	AUC	Brier score
Internal validation cohort						
SVM	0.682	0.660	0.732	0.694	0.777	0.255
RF	0.788	0.772	0.810	0.790	0.853	0.194
GBDT	0.767	0.755	0.782	0.768	0.850	0.199
AdaBoost	0.750	0.745	0.750	0.747	0.831	0.215
XGBoost	**0.797**	0.777	**0.824**	**0.800**	0.852	**0.193**
LightGBM	0.795	**0.786**	0.803	0.794	**0.861**	0.194
CatBoost	0.785	0.772	0.799	0.785	0.860	0.195
NGBoost	0.752	0.746	0.754	0.750	0.829	0.202
EL model	**0.842**	**0.830**	**0.839**	**0.835**	**0.898**	**0.137**
EL model*	**0.804**	0.782	**0.843**	**0.811**	**0.866**	**0.143**
External validation cohort						
EL model*	0.802	0.781	0.805	0.793	0.851	0.161

* presented the EL model that used the top 7 features clinical features.

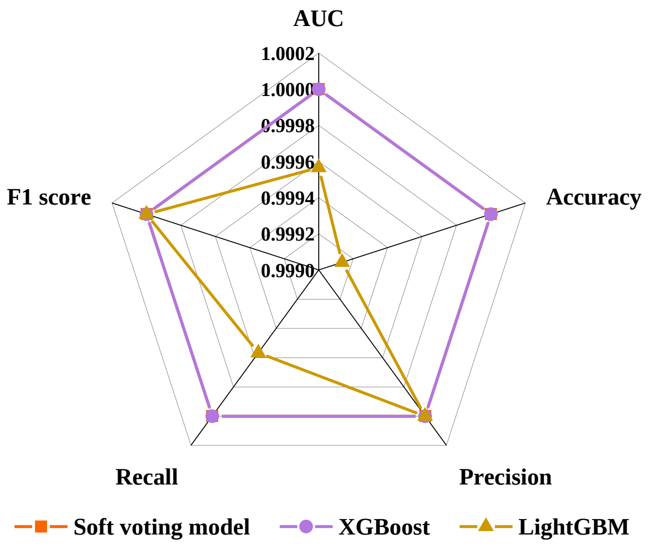

Fig. S2. Training results of EL model, XGBoost and LightGBM.

Text S2 The composite score of feature importance obtained by considering Gini impurity and SHAP values

Due to inconsistent results derived from the Gini impurity and SHAP methods, important features that occurred three or more times in **Fig. 6** were re-scored. The score was assigned according to **Table S2** for each occurrence of a feature and then was summed to obtain the final composite score. The composite score represented a more comprehensive feature importance analysis, which not only takes into account the focus of the different basic models in the soft voting ensemble, but also the emphasis of the different model interpretation methods. For example, HCO_3^- occurred only in Fig. 6a, c and d, but was all ranked in the top. In contrast, RR occurred in all Fig. 6a, b, c and d, but was ranked low in all of them. In this case, the number of occurrences cannot be used as an criterion to deem that RR is more important than HCO_3^-. The composite score provided a more fair comparison, with HCO_3^- scoring 20+20+19=59 and RR scoring 3+12+7+2=24, indicating that HCO_3^- had a more significant influence on outcome. Since our model is ensemble by XGBoost and LightGBM, the composite score could provide a more appropriate and valuable feature importance analysis.

Table S4. Feature importance scoring based on Gini impurity and SHAP method.

The feature importance ranking in Gini impurity or SHAP method	Feature importance score	The feature importance ranking in Gini impurity or SHAP method	Feature importance score
1	20	11	10
2	19	12	9
3	18	13	8
4	17	14	7
5	16	15	6
6	15	16	5
7	14	17	4
8	13	18	3
9	12	19	2
10	11	20	1

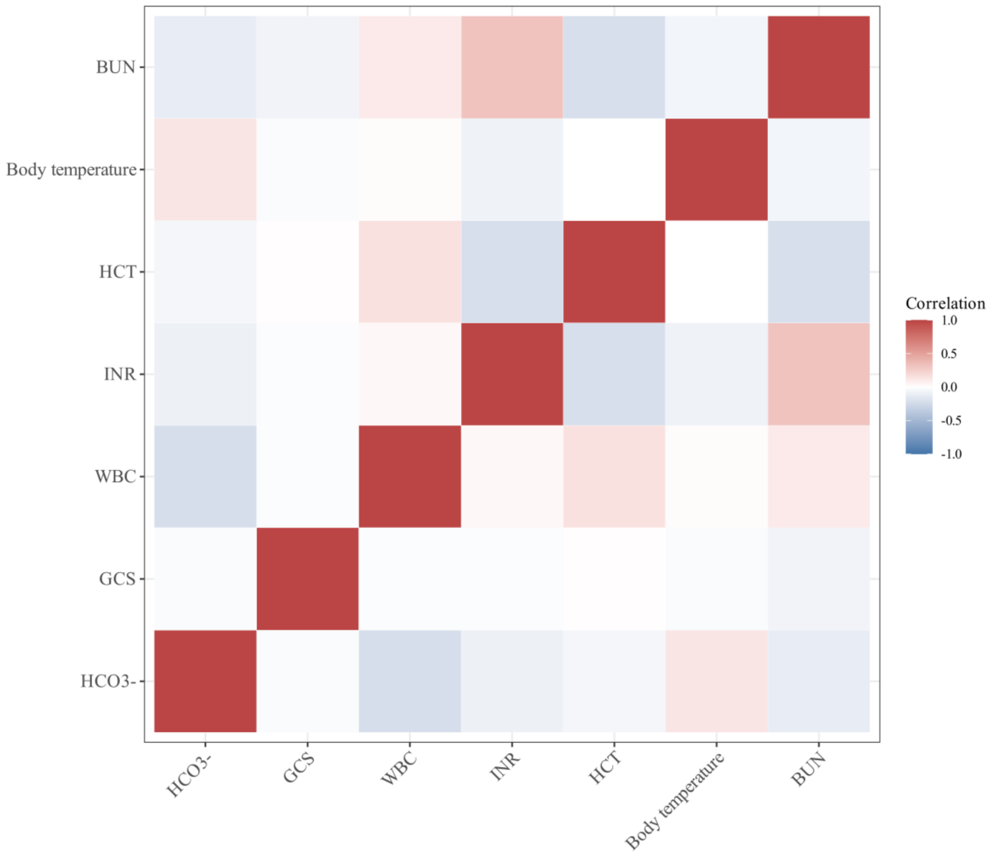

Fig. S3. Pearson correlation analysis for HCO_3^-, GCS, WBC, INR, HCT, body temperature and BUN to exclude multicollinearity.

Table S5. Baseline characteristics of the patients between mimic-iv database and model failure prediction.

Feature	MIMIC-IV database	Model failure prediction	P value
HCO3-	21.48 (4.55)	22.00 (3.91)	0.311
GCS	15.00 (14.60, 15.00)	14.50 (14.40, 15.00)	0.694
WBC	12.75 (9.20, 17.08)	14.24 (10.77, 16.51)	0.331
INR	1.30 (1.15, 1.60)	1.55 (1.20, 1.61)	0.912
HCT	33.65 (28.70, 39.04)	33.50 (29.91, 36.73)	0.333
Body temperature	36.74 (36.41, 37.08)	36.68 (36.47, 37.01)	0.185
BUN	30.10 (22.16)	30.27 (18.74)	0.749
